# Gartland type III supracondylar humerus fractures: outcome and complications as related to operative timing and pin configuration

**DOI:** 10.1007/s11832-014-0624-x

**Published:** 2014-11-08

**Authors:** Matthew D. Abbott, Lucas Buchler, Randall T. Loder, Christine B. Caltoum

**Affiliations:** 1 Department of Orthopaedic Surgery, University of Michigan School of Medicine, Ann Arbor, MI USA; 2 Department of Orthopaedic Surgery, Indiana University School of Medicine, Indianapolis, IN USA; 3 Department of Pediatric Orthopaedic Surgery, C.S. Mott Children’s Hospital, University of Michigan School of Medicine, 1540 East Medical Center Drive, Ann Arbor, MI 48109 USA

**Keywords:** Supracondylar, Humerus, Pediatric, Trauma

## Abstract

**Purpose:**

Supracondylar fractures of the humerus are the most common fracture of the elbow in children. The purpose of this study was to evaluate, in terms of outcomes and complications, Gartland type III pediatric supracondylar humerus fractures treated at a pediatric level-one trauma center over a 7-year period, specifically addressing the impact of time to surgery on the incidence of complications and conversion to open reduction.

**Methods:**

We retrospectively reviewed 297 pediatric patients that sustained a closed Gartland type III supracondylar humerus fracture treated between December 2004 and December 2011. The time to the operating room was calculated from the medical records for each patient. The outcome measures evaluated were operative time, conversion to open procedure, and perioperative and postoperative complications.

**Results:**

In our study, there were 30 complications in 25 children (8.4%). Conversion to open reduction occurred in 28 children (9.4%). The time from the emergency department to the operating room was not significantly correlated with increased complications, increased operative time, or conversion to open reduction (*p* > 0.05). Crossed pinning resulted in an increased risk of overall complications [odds ratio (OR) = 2.6] and iatrogenic nerve injuries (OR = 9.3). Complications also occurred more commonly in boys (OR = 3.3) and in older patients (*p* = 0.0069)

**Conclusions:**

We found no significant correlation between the time to surgery and complications, operative time, or need for open reduction. These findings support the trend of treating Gartland type III supracondylar humerus fractures in a less urgent manner. In addition, our study supports the concept that cross pinning leads to more complications than lateral pinning, including an 8-fold increase in iatrogenic nerve injury.

## Introduction

 Supracondylar fractures of the humerus are the most common fracture of the elbow in children. The majority of these fractures (96–98 %) are extension-type fractures [1–4]. Gartland originally described a classification for extension-type supracondylar humerus fractures, dividing them into three types: type I is non-displaced, type II is displaced with an intact posterior cortex, and type III is displaced without cortical contact [2, 5]. The current preferred treatment for Gartland type III fractures consists of attempted closed reduction and percutaneous pinning [3, 6–8].

The focus of many recent research studies on the treatment of these fractures has been pin configuration. Biomechanical testing has demonstrated a theoretical advantage of both medial and lateral cross pinning; however, these initial findings have not translated into clinical results [9–11]. The concern for iatrogenic ulnar nerve injury during the placement of a medial pin discourages this configuration. Slobogean et al. conducted a systematic review focusing on iatrogenic ulnar nerve injury due to the placement of a medial pin and concluded that the number needed to harm was 28 patients [12].

Until the late 1990s, it was believed that displaced pediatric supracondylar humerus fractures required emergent surgical intervention or skeletal traction. The theoretical advantage proposed that this would lead to a decrease in perioperative complications, including iatrogenic nerve palsies, compartment syndrome, and conversion to an open reduction [6, 8, 13, 14]. In recent years, many adult trauma hospitals have decreased night-time on-call orthopedic surgery without affecting patient outcomes by allowing dedicated trauma operative time during the day [15, 16]. In a similar fashion, many pediatric studies have been performed questioning the necessity of urgent night-time intervention in Gartland type III supracondylar humerus fractures. In 1999, Iyengar et al. published a retrospective review comparing early versus delayed (greater than 8 h following fracture) treatment of 58 patients with type III fractures and showed no difference in terms of clinical results or perioperative complications, including conversion to open reduction [8]. Multiple studies followed showing similar results [6, 14, 17]. Conversely, studies by Walmsley et al. and Yildirim et al. showed that delayed intervention resulted in an increase in open reduction but no other perioperative complications [7, 13]. With regards to operative delay leading to open reduction, there is literature supporting both arguments, resulting in a lack of conclusive agreement.

The purpose of this study was to evaluate the outcomes and complications of Gartland type III pediatric supracondylar humerus fractures treated at a pediatric level-one trauma center over a 7-year period, specifically addressing the impact of the time to surgery on the incidence of postoperative complications and conversion to an open reduction.

## Materials and methods

All children with closed type III supracondylar humerus fractures admitted and treated between January 2004 and December 2011 at the author’s institution were identified. Patients with open fractures and/or vascular injury were excluded. The medical charts and operative records were retrospectively reviewed. The age at surgery, gender, weight, and initial examination findings of skin tenting, ecchymosis, and preoperative neurologic deficit was collected. The time from the emergency department to the operating room and the time from injury (if available) to the operating room were calculated. Operative records were reviewed for total operative time, number of K-wires used, K-wire configuration, and open or closed reduction. The outcome measures consisted of any unplanned return to the operating room, conversion to open reduction, pin site infection, iatrogenic nerve injury, compartment syndrome, trochlear avascular necrosis, and length of stay, defined as the time following surgery to discharge. This study was approved by our local Institutional Review Board.

During this time period, there were 297 children who sustained a supracondylar humerus fracture treated by closed or open reduction and percutaneous pinning by 11 different surgeons (Table 1). There were 149 boys and 148 girls, with an average age of 5.8 ± 2.2 years. The majority of injuries occurred as a result of a fall (46.8 %) or playground injury (35.5 %). Lateral pin configuration was used in 206 children, with an average of 3.0 pins; cross pins were used in 91 children, with an average of 2.9 pins (Table 2). The pin configuration was determined solely by the discretion of the treating surgeon based on a multitude of factors, including fracture pattern and configuration preference. The average length of follow up was 3.1 months.

**Table 1:** Demographics

	Overall	%
Patients	297	
Age (years)	5.8 ± 2.2	
Gender		
Male	149	50.2
Female	148	49.8
MOI		
Fall	139	46.8
Playground	105	35.4
Vehicle	25	8.4
Sports	13	4.4
Trampoline	6	2.0
Blunt	4	1.3
Unknown	5	1.7
Preoperative neural deficits	46	15.5

**Table 2:** Operative and postoperative data

	Number	%
Pin configuration		
Lateral	206	69.4
Cross	91	30.6
Complications		
Iatrogenic nerve injury	14	4.7
Pin site infection	8	2.7
Return to operating room	6	2.0
Compartment syndrome	1	0.3
AVN	1	0.3
Total	30 (25 patients)	8.4
Length of follow up (months)	30.1	

### Statistical analysis

Continuous data are reported as the mean ± 1 standard deviation. Discrete data are reported as frequencies and percentages. Statistical analyses between continuous data were performed with the non-parametric Mann–Whitney *U*-test; differences between discrete data were determined by Fisher’s exact test. Stepwise, backwards binary logistic regression analyses were performed to determine predictors [odds ratios (ORs) with 95 % confidence intervals] of variables for a particular outcome. The variables entered into the analyses were those having an appropriate *p*-value from the bivariate analysis. Entry into the model was set at *p* = 0.20 and removal at *p* = 0.05. Statistical analyses were performed with SYSTAT 10 software™ (Chicago, IL, 2000). For all statistical analyses, a *p* < 0.05 was considered statistically significant.

## Results

Conversion to open reduction occurred in 28 patients (9.4 %) (Table 3). Those having an open reduction were older; the child’s gender, time from the emergency department to the operating room, and time from injury to the operating room were not statistically different. There were 30 complications which occurred in 25 children (8.4 %). The complications included 14 iatrogenic nerve injuries (4.7 %), eight pin site infections (2.7 %), six unplanned returns to the operating room (2.0 %), one compartment syndrome (0.3 %), and one avascular necrosis (0.3 %). The six children with unplanned returns to the operating room consisted of two for loss of reduction, one for compartment syndrome, one irrigation and debridement for a septic elbow, one for neurolysis, and one distal humeral osteotomy for range of motion issues. Those with complications were older, more frequent in boys, and had undergone cross pinning (Table 4). The times from the emergency department to the operating room and injury to the operating room were not statistically different. Iatrogenic nerve injury occurred in 14 patients (Table 5). Those with an iatrogenic nerve injury were older and had undergone cross pinning. There were 46 children with 46 preoperative neural deficits. Those with deficits were older and more frequently associated with a postoperative pin site infection.

**Table 3:** Conversion to open reduction

	Yes	No	*p*-Value
Patients	28	269	
Age (years)	6.8 ± 2.5	5.7 ± 2.2	0.034
Emergency department to operating room (h)	10.1 ± 5.5	8.3 ± 5.6	0.081
Injury to operating room (h)	15.5 ± 5.6	13.1 ± 6.3	0.06
Gender			
Boys	15 (10 %)	134	0.71
Girls	13 (8.8 %)	135

**Table 4:** Complications

	Yes	No	*p*-Value
Patients	25	272	
Age (years)	6.9 ± 1.9	5.7 ± 2.3	0.0069
Emergency department to operating room (h)	9.1 ± 5.2	8.4 ± 5.6	0.43
Injury to operating room (h)	12.3 ± 6.0	13.4 ± 6.3	0.62
Gender			
Boys	18 (12.1 %)	131	0.023
Girls	7 (4.7 %)	141	
Pinning			
Cross	13 (14.3 %)	78	0.016
Lateral	12 (5.8 %)	194	
No. of pins used	3.1 ± 0.6	3.0 ± 0.5	0.11
Operating room time (min)	46.1 ± 37.2	37.6 ± 21.4	0.96

**Table 5:** Iatrogenic nerve injury

	Yes	No	*p*-Value
Patients	14	283	
Age (years)	7.0 ± 1.6	5.7 ± 2.3	0.016
Pinning			
Cross	11 (12.1 %)	80	0.0001
Lateral	3 (1.5 %)	203	
No. of pins used	3.1 ± 0.7	3.0 ± 0.5	0.18
Operating room time (min)	47.1 ± 36.4	37.9 ± 22.3	0.56

For the purposes of logistic regression analysis, the median age was 5.8 years, which allowed us to create two age groups; those >5.8 years old, who were designated as older, and those <5.8 years old, who were designated as younger. Those with a complication were more frequently male [OR 3.3 (1.3, 8.6)] and had been cross pinned [OR 2.6 (1.1, 6.0)]. Those converted to an open reduction were older [OR 3.1 (1.3, 7.7)] and more likely to have been cross pinned [OR 4.6, (2.0, 10.5)]. The overall risk of complications was higher in boys [OR 3.3 (1.3, 8.6)] and those with cross pins [OR 2.6 (1.1, 6.0)]. Those having an iatrogenic nerve injury were more frequently cross pinned [OR 9.3 (2.6, 34.3)].

## Discussion

Pediatric supracondylar humerus fractures have been the focus of significant research due to the common nature of such injuries. With this vast array of literature, conflicting ideas and findings have resulted in a lack of conclusive agreement regarding surgical timing and pin configuration. In addition, there is a wide severity spectrum of supracondylar humerus fractures even within Gartland type III fractures; this makes the literature difficult to interpret and utilize. Ideally, surgeons should be able to differentiate severe and non-severe Gartland type III supracondylar humerus fractures to aid in determining which patients are at higher risk for complications and require open reduction.

In our study, we found no significant correlation between the time from the emergency department or the injury to the operating room and complications, need for open reduction, or operative time, similar to previous studies [6, 8, 14, 17]. In recent years, adult orthopedic trauma surgeons have begun to limit middle-of-the-night urgent surgeries seemingly without affecting patient outcomes [15, 16]. Likewise, in pediatric orthopedics, a similar trend has begun to emerge. These findings support that current trend toward daytime treatment of Gartland type III supracondylar humerus fractures rather than night-time urgent surgery. At our institution, we feel comfortable treating type III supracondylar humerus fractures as the first morning case; however, severe injuries (e.g., vascular injuries, nerve deficits, severe swelling with ecchymosis) are still treated in a more urgent fashion.

Although the time to surgery did not have an effect on the complication rate, we did note an increased rate of complications due to pin configuration. In our study, cross pinning led to an 8-fold increase in iatrogenic nerve injury. Historically, the pin configuration of pediatric supracondylar humerus fractures has been a popular debate. The majority of the literature has shown an increased risk of iatrogenic nerve injury when cross pinning is performed, although some studies disagree [3, 12, 17–20]. Our results indicate that lateral pinning is a much safer procedure than cross pinning in terms of complications (5.8 vs. 14.3 %), which has been supported in the literature.

In addition to pin configuration, older patients and males in our study were at higher risk for complications. Older patients presented with more fractures that resulted in an increased likelihood of preoperative nerve deficit, greater risk of complications, and increased rate of conversion to open reduction. Fletcher et al. recently published similar findings of increased severity with regards to age [21]. They hypothesized that this was a result of higher-energy injury mechanism. The study by Farnsworth et al. showing higher-energy mechanism after the age of 4 years would support this claim [22]. Males had an overall increased risk of complications in our study. It is unclear as to the etiology of this gender finding; however, it is possible that this is also due to a higher-energy injury mechanism. Unfortunately, the retrospective nature of this study and lack of detailed injury information makes evaluating the mechanism of injury as a possible etiology problematic. To our knowledge, there has not been a study published directly connecting the severity of fracture and gender.

There are many limitations to our study. First, it is retrospective in nature. Second, there are multiple surgeons included in this study, which leads to variability in operative timing and technique. In addition, the inclusion of multiple surgeons may add bias in the decision to convert to open reduction, as the ultimate decision is likely to be different from surgeon to surgeon.

In summary, treating Gartland type III supracondylar humerus fractures in a non-urgent fashion did not increase the risk of complications or conversion to open reduction in our study population. While we believe that many of these fractures can be treated the following morning, there are still some specific urgent indications. In addition, our study supports the concept that cross pinning leads to more complications than lateral pinning.

## References

[1] Carmichael KD, Joyner K (2006). Quality of reduction versus timing of surgical intervention for pediatric supracondylar humerus fractures. Orthopedics.

[2] Mallo G, Stanat SJC, Gaffney J (2010). Use of the Gartland classification system for treatment of pediatric supracondylar humerus fractures. Orthopedics.

[3] Babal JC, Mehlman CT, Klein G (2010). Nerve injuries associated with pediatric supracondylar humeral fractures: a meta-analysis. J Pediatr Orthop.

[4] White L, Mehlman CT, Crawford AH (2010). Perfused, pulseless, and puzzling: a systematic review of vascular injuries in pediatric supracondylar humerus fractures and results of a POSNA questionnaire. J Pediatr Orthop.

[5] Gartland JJ (1959). Management of supracondylar fractures of the humerus in children. Surg Gynecol Obstet.

[6] Mehlman CT, Strub WM, Roy DR (2001). The effect of surgical timing on the perioperative complications of treatment of supracondylar humeral fractures in children. J Bone Joint Surg Am.

[7] Yildirim AO, Unal VS, Oken OF (2009). Timing of surgical treatment for type III supracondylar humerus fractures in pediatric patients. J Child Orthop.

[8] Iyengar SR, Hoffinger SA, Townsend DR (1999). Early versus delayed reduction and pinning of type III displaced supracondylar fractures of the humerus in children: a comparative study. J Orthop Trauma.

[9] Brauer CA, Lee BM, Bae DS (2007). A systematic review of medial and lateral entry pinning versus lateral entry pinning for supracondylar fractures of the humerus. J Pediatr Orthop.

[10] Kocher MS, Kasser JR, Waters PM (2007). Lateral entry compared with medial and lateral entry pin fixation for completely displaced supracondylar humeral fractures in children. A randomized clinical trial. J Bone Joint Surg Am.

[11] Skaggs DL, Cluck MW, Mostofi A (2004). Lateral-entry pin fixation in the management of supracondylar fractures in children. J Bone Joint Surg Am.

[12] Slobogean BL, Jackman H, Tennant S (2010). Iatrogenic ulnar nerve injury after the surgical treatment of displaced supracondylar fractures of the humerus: number needed to harm, a systematic review. J Pediatr Orthop.

[13] Walmsley PJ, Kelly MB, Robb JE (2006). Delay increases the need for open reduction of type-III supracondylar fractures of the humerus. J Bone Joint Surg Br.

[14] Sibinski M, Sharma H, Bennet GC (2006). Early versus delayed treatment of extension type-3 supracondylar fractures of the humerus in children. J Bone Joint Surg Br.

[15] Wixted JJ, Reed M, Eskander MS (2008). The effect of an orthopedic trauma room on after-hours surgery at a level one trauma center. J Orthop Trauma.

[16] Bhattacharyya T, Vrahas MS, Morrison SM (2006). The value of the dedicated orthopaedic trauma operating room. J Trauma.

[17] Garg S, Weller A, Larson AN (2014). Clinical characteristics of severe supracondylar humerus fractures in children. J Pediatr Orthop.

[18] Skaggs DL, Hale JM, Bassett J (2001). Operative treatment of supracondylar fractures of the humerus in children. The consequences of pin placement. J Bone Joint Surg Am.

[19] Lyons JP, Ashley E, Hoffer MM (1998). Ulnar nerve palsies after percutaneous cross-pinning of supracondylar fractures in children’s elbows. J Pediatr Orthop.

[20] Edmonds EW, Roocroft JH, Mubarak SJ (2012). Treatment of displaced pediatric supracondylar humerus fracture patterns requiring medial fixation: a reliable and safer cross-pinning technique. J Pediatr Orthop.

[21] Fletcher ND, Schiller JR, Garg S (2012). Increased severity of type III supracondylar humerus fractures in the preteen population. J Pediatr Orthop.

[22] Farnsworth CL, Silva PD, Mubarak SJ (1998). Etiology of supracondylar humerus fractures. J Pediatr Orthop.

